# T138. ACOUSTIC PATTERNS IN SCHIZOPHRENIA: A SYSTEMATIC REVIEW AND META-ANALYSIS

**DOI:** 10.1093/schbul/sby016.414

**Published:** 2018-04-01

**Authors:** Alberto Parola, Arndis Simonsen, Vibeke Bliksted, Riccardo Fusaroli

**Affiliations:** 1 University of Turin; 2 Aarhus University

## Abstract

**Background:**

Individuals with schizophrenia are characterized as presenting atypical voice patterns: poverty of speech, increased pauses, distinctive pitch (mean and variability). Voice atypicalities may play a role in the social impairment experienced by patients, and could constitute a window into motor, cognitive, emotional and social components of the disorder. Indeed, they have already been generally associated with negative symptoms. However, the state of the evidence for atypical voice patterns and their relation to clinical features is uncertain. Studies using clinical rating scales indicate that voice alterations are severe across many voice properties. In contrast, quantitative acoustic studies seem to have found less robust and more variable results limited to specific features. We therefore systematically reviewed the literature quantifying acoustic patterns in schizophrenia, and performed a meta-analysis of the evidence. We aimed at identifying evidence for acoustic markers of schizophrenia and its clinical features, needs for further research and barriers to collective advancements on these issues.

**Methods:**

We adopted the “PRISMA Statement” guidelines for transparent reporting of a systematic review. The literature search was conducted on Pubmed and Google Scholar (details and pre-registration at https://goo.gl/H1yDpm). Study selection was conducted according to the following inclusion criteria: (a) empirical study, (b) quantification of acoustic features in the vocal production of participants with schizophrenia, (c) sample including at least two individuals with schizophrenia, (d) inclusion of a comparison group, or an assessment of variation in acoustic features in relation to severity of clinical features. We identified 54 studies as eligible for inclusion and contacted all authors to obtain missing estimates and individual-level data, where possible. 34 studies availed enough information to be included in a meta-analysis. The meta-analysis consisted of mixed effects regression models, one per each relevant acoustic feature.

**Results:**

Of the 37 authors contacted, 59% responded and 5% provided at least some of the requested data. Chief reasons of denials were: i) data loss (n = 8), ii) effort required (n = 5), iii) ethical concerns with data sharing (n = 1). On the results available we found significant meta-analytic effects of schizophrenia in percentage of spoken time (n = 6, d = -1.16, 95% CIs: -2.06 -0.27) and proportion of pauses (n = 5, d = 0.56, 95% CIs: 0.15 0.96). After controlling for influential studies, we found significant differences also in pitch mean (n = 5, d = 0.40, 95% CIs: 0.12 0.68) and pitch variability (n = 6, d = -0.46, 95% CIs: -0.70 -0.23). No effects were found for pause duration (n = 7), speech rate (n = 9), speech duration (n = 5) and pitch intensity (n = 5). We found evidence for publication bias for studies investigating pause duration and pitch variability.

Key concerns on the meta-analysis are: i) small sample sizes, ii) heterogeneity of task and acoustic processing methods, iii) lack of demographic and clinical individual-level data necessary to control for confounds (e.g. medication and relation to clinical features).

**Discussion:**

We found clear effects of increased pause behavior in schizophrenia and less clear effects of pitch. However, the magnitude of these abnormalities is limited and contrast with the large effect sizes reported by studies using clinical rating scales. Future research should focus on larger sample sizes, systematic assessment of multiple acoustic features and multiple speech tasks, standardized acoustic processing methods, and individual level data available. More reflection is needed on how to make data sharing possible within privacy and ethical constraints.

